# Anti-Inflammatory Effects of Peripheral Dopamine

**DOI:** 10.3390/ijms241813816

**Published:** 2023-09-07

**Authors:** Shaun C. Moore, Pedro A. S. Vaz de Castro, Daniel Yaqub, Pedro A. Jose, Ines Armando

**Affiliations:** Division of Kidney Diseases and Hypertension, Department of Medicine, The George Washington School of Medicine and Health Sciences, Washington, DC 20037, USA; scmoore@gwu.edu (S.C.M.); pcastro@email.gwu.edu (P.A.S.V.d.C.); dyaqub@gwu.edu (D.Y.); pjose@mfa.gwu.edu (P.A.J.)

**Keywords:** immune cells, signaling pathways, kidney, mesenteric organs, pro-inflammatory factors

## Abstract

Dopamine is synthesized in the nervous system where it acts as a neurotransmitter. Dopamine is also synthesized in a number of peripheral organs as well as in several types of cells and has organ-specific functions and, as demonstrated more recently, is involved in the regulation of the immune response and inflammatory reaction. In particular, the renal dopaminergic system is very important in the regulation of sodium transport and blood pressure and is particularly sensitive to stimuli that cause oxidative stress and inflammation. This review is focused on how dopamine is synthesized in organs and tissues and the mechanisms by which dopamine and its receptors exert their effects on the inflammatory response.

## 1. Introduction

In addition to the central nervous system, dopamine (DA) is produced locally in several peripheral organs and in different types of cells and influences numerous functions, including gastrointestinal motility, metabolic homeostasis, hormone release, sodium balance, and blood pressure [1,2,3]. In the periphery, under normal conditions, very little DA is released from the adrenal medulla or the sympathetic nerves into the circulation; circulating concentrations of DA in the free form, ranging from 0 to 30 pg/mL (195.8 pmol/L) in humans [4], are too low to have any physiological effect. However, significant amounts of DA are produced by organs other than the brain. In humans and laboratory animals, at least 90–95% of DA in the plasma circulates in the sulfoconjugated form. DA is sulfoconjugated before entering the bloodstream and its formation is dependent on the intracellular synthesis of L-DOPA and DA in non-adrenergic cells and in the uptake of circulating L-DOPA [5]. The lung, mesentery, and other organs and cell types contribute to the total body production and metabolism of DA [6]. Peripheral production of DA results in urine concentrations of DA and its metabolites that are higher than those of norepinephrine (NE) and its metabolites, underlying the importance of the dopaminergic system and supporting the notion that DA in the periphery acts as an autocrine/paracrine hormone that is rapidly inactivated by sulfoconjugation [5,7].

In addition to the functions of DA mentioned above, DA and dopaminergic drugs that bind to their receptors have been demonstrated to regulate the immune response as well as the inflammatory reaction [8]. Both in vitro and in vivo studies suggest that DA can suppress the inflammatory reaction. Retained during evolution, the peripheral dopaminergic system is beginning to be recognized as an endogenous anti-inflammatory system. DA acts via two subfamilies of G-protein-coupled receptors, namely D1-like (D1R and D5R) and D2-like (D2R, D3R, and D4R) receptors. All the DA receptor subtypes are differentially expressed in tissues depending on their physiological function. The mechanisms underlying these effects and actions are starting to be recognized and emerging evidence suggests that they can be tissue-specific. This review focuses on the effects and mechanisms of peripheral DA on inflammation.

### 1.1. Dopamine and Immunomodulation

Several studies have shown that DA functions as an immunomodulatory regulator and that this regulation is an important part of the healthy immune function. More recent studies have reported DA-induced changes in the functions of lymphocytes, macrophages, monocytes, and neutrophils. All types of immune cells produce dopamine at low levels and dopamine receptors are expressed on T cells, B cells, neutrophils, eosinophils, natural killer (NK) cells, dendritic cells, macrophages, microglia, and monocytes [9,10,11,12]. Acting in an autocrine/paracrine manner, DA modulates the functions of immune cells through D1-like and D2-like receptors. These receptors have been reported to regulate the activation, inhibition, and proliferation of immune cells and their functions [13,14,15,16,17,18,19,20,21]. The ability of DA to inhibit the production of reactive oxygen species by human polymorphonuclear leukocytes and their migration is dependent on D1-like receptors’ activation, in particular the D5R [17].

The administration of dopexamine, a synthetic analog of dopamine with β2 adrenergic properties, in patients undergoing cardiopulmonary bypass reduces circulating TNFα levels and leukocyte count [22]. Dopexamine also decreases the number of leukocytes adhering to the vascular endothelium and plasma TNFα levels in a rat model of experimental sepsis [23]. DA also inhibits the activation of T cells, resulting in the downregulation of their proliferation and secretion of the cytokines IL-2, IL-6, IL-4, IFN-c, and IL-4 [24]. However, the D1-like receptor antagonist SCH23390 has been shown to attenuate Th17-mediated immune diseases such as experimental autoimmune encephalomyelitis [25], autoimmune diabetes in non-obese diabetic mice [26], and nephrotoxic serum nephritis [27]. These findings indicate that the effects of DA on T cells are dependent on the concentrations of DA or DA agonists present, the type and subtypes of T cells, and more importantly on the state of T cell activation [14,15,16,18,19,20]. In peritoneal macrophages, DA inhibits the lipopolysaccharide (LPS)-stimulated production of IL-12p40 and increases the production of the anti-inflammatory cytokine IL-10 [28]. However, in contrast to these inhibitory effects of DA on inflammation in these cells, dopexamine, a synthetic analog of dopamine with D1-and D2-like receptor and β2-adrenergic properties increased circulating TNFα, soluble TNF receptor, and leucocyte count in humans [22]. Nevertheless, there is agreement that DA inhibits NLRP3 inflammasome activation in macrophages. In LPS-treated bone marrow-derived macrophages, treatment with DA at high concentrations, before a challenge with nigericin, an NLRP3 stimulant, negatively regulates the NLRP3 inflammasome activation through D1R signaling via cyclic adenosine monophosphate (cAMP), which promotes the ubiquitination and degradation of the inflammasome [13]. Moreover, DA at high concentrations (>100 μM) suppresses pro-inflammatory mediators (IL-1β and IL-18 but not TNFα) and the NLRP3 activation in macrophages stimulated with LPS [29]. Furthermore, DA and D1R signaling ameliorates, in vivo, monosodium urate crystal-induced peritoneal inflammation, neurotoxin-induced neuroinflammation, and LPS-induced systemic inflammation all inflammatory pathologies that mediated by the NLRP3 inflammasome, although these effects are only observed at doses of DA higher than 100 μM [13] that may activate receptors other than DA, such as α1, α2, and β-adrenergic receptors [2].

The role of D2-like receptors, relative to D1-like receptors, on immune cells appears to be less controversial. Either D2-like agonist- or cell-specific D2R activation attenuates inflammation in rodent models of sepsis or chronic neuroinflammation [10,11]. Three D2-like receptor agonists, pramipexole (D3R > D2R), bromocriptine (D2R = D3R > D4R), and pergolide (D3R > D2R > D4R > D5R > D1R), have also been shown to possesses significant anti-inflammatory activity in several models of inflammation in rodents, reducing tissue injury, neutrophil infiltration, and subcutaneous edema [30,31]. D2-like receptors appear to be more important than D1-like receptors in regulating T cells. In T lymphocytes stimulated with concanavalin A, quinpirole, a D2-like receptor (D3R = D4R > D2R) agonist, upregulated the expression of specific transcription factors and cytokines of Th2 and Treg but downregulated the expression of specific transcription factors and cytokines of Th1 and Th17, promoting the differentiation of T lymphocytes to an anti-inflammatory phenotype [32]. In isolated bone marrow-derived macrophages, treatment with the D2R agonist quinpirole inhibited M1 macrophage polarization, decreased NADPH oxidase-mediated oxidative stress and NF-κB, and it also decreased the activation of the NLRP3 inflammasome, effects that were lost after specific deletion of the D2R in bone marrow macrophages [33]. However, in both immortalized and primary macrophages in culture, pretreatment with the D2R antagonist haloperidol (D4R > D2R = D3R = D1R > D5R) inhibited the activation of NF-κB induced by LPS and decreased CD80 expression and the secretion of pro-inflammatory cytokines (IL-1β, IL-6, and IL-12p40) [34]. These effects (CD80 and IL-6) were also attenuated by a different D2-like receptor antagonist, L750.667, that may be selective to D4R [35]. Meanwhile, a D1-like receptor antagonist had no effect, suggesting actions via D2-like receptors [34]. For an in-depth review of the role of dopamine on the immune system please see ref. [8].

### 1.2. Anti-Inflammatory Effects of Dopamine in Peripheral Organs, Tissues, and Cells

Inflammation is a complex process that is necessary to defend organisms from pathogens or injury. However, it becomes harmful when it occurs in the absence of noxious stimuli or is not controlled in a timely manner. In organs and tissues, inflammation depends on two major factors, the intrinsic response of specific cells in the tissue and the infiltration of immune cells into those tissues. The interrelations of these factors can be tissue-specific.

#### 1.2.1. Dopamine in Abdominal Organs

**Figure 1:**
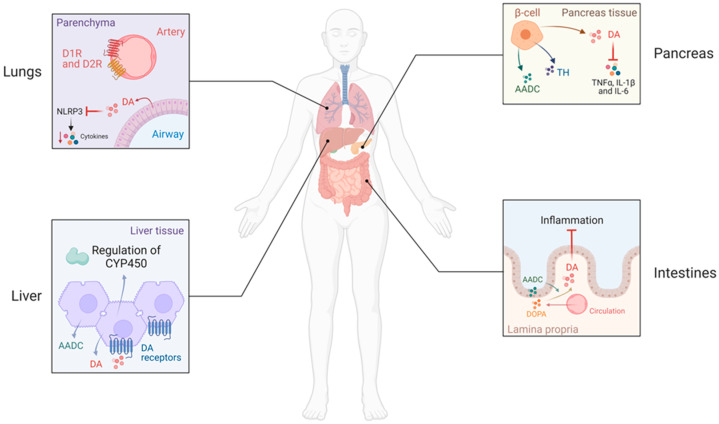
Peripheral dopamine (DA) production and actions. In the lung, alveolar type II epithelial cells can synthesize DA, which attenuates lung tissue injury, neutrophil infiltration, and inhibits inflammatory cytokine response through inhibition of NLRP3-signaling pathways. DA may also modulate ventilation by acting on dopamine D1 (D1R) and D2 (D2R) receptors in the arteries of the lung. In the liver, aromatic l-amino acid decarboxylase (AADC) is expressed, and DA is produced by hepatocytes. D2R can modulate the regulation of components of the cytochrome P450 (CYP450). Moreover, DA may protect the liver from acute injury. In the pancreas, DA is synthesized mainly in β-cells that intracellularly express TH and AADC and regulate insulin production in these cells. DA reduces the increased expression of inflammatory cytokines (TNFα, IL-1β, and IL-6) induced by cholecystokinin in acute experimental pancreatitis. In the intestines, DA is abundant in the mucosal cell layer; the epithelial cells of the intestinal mucosa are rich in AADC and circulating DOPA is taken up to produce DA. DA reduces the inflammation in human inflammatory bowel disease.

Abdominal organs (mesentery, gastrointestinal tract, spleen, and pancreas) produce a substantial portion of the total DA produced in the body [36,37,38]. Not only can some enteric neurons synthetize and release DA [36,37] but they can also stimulate tyrosine hydroxylase (TH), the enzyme that synthesizes DOPA from tyrosine. Other enzymes in the DA synthetic pathway are expressed in many cells in the abdominal organs (Figure 1). In human and experimental animals, DA synthesis has been reported in parietal cells and other epithelial cells in the stomach [38].

DA is abundant in the mucosal cell layer of the intestine; the epithelial cells of the intestinal mucosa have abundant aromatic L-amino acid decarboxylase (AADC) and are capable of the uptake of circulating DOPA to produce DA, particularly in the jejunum. In the gastrointestinal tract, DA has marked anti-inflammatory properties mediated by the suppression of interleukins (IL-1β, IL-6) and TNFα, as well as inhibition of the activation of the NLRP3 inflammasome, that is mediated by the D1R [13] and D2R [39]. The D2-like receptor agonist, bromocriptine (D2R = D3R > D4R), ameliorated a drug-induced inflammatory bowel disease and decreased its mortality, whereas treatment with the D2-like receptor antagonist domperidone (D2R > D3R), worsened the condition and increased the mortality rate [40]. By contrast, D3R deficiency attenuated gut inflammation in mice caused by dextran sodium sulfate by decreasing IL-10 production and gut growth [41], indicating that dopamine exerts its protective effect in drug-induced gut inflammation via the D2R. The concentration of DA in the inflamed mucosa of ulcerative colitis and Crohn’s disease patients was markedly lower than in controls. This resulted in significant reductions in DA/L-DOPA tissue ratios, which is a rough measure of aromatic L-amino acid decarboxylase activity [42]. Treatment with D2-like receptor antagonists quinpirole (D3R = D4R = D2R) or cabergoline (D2R = D3R) ameliorated the colonic lesion in two animal models of ulcerative colitis via downregulation of AKT phosphorylation and c-Src [43].

**Figure 2:**
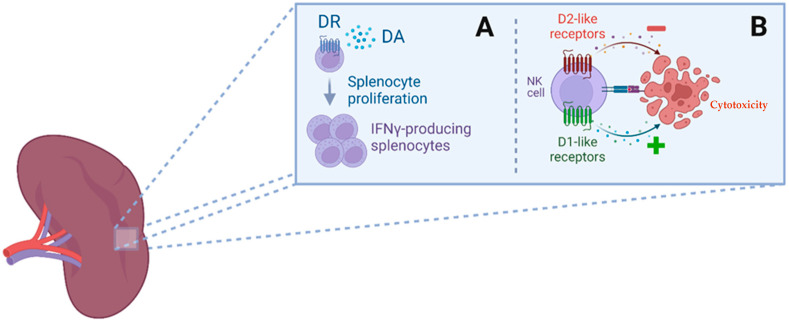
Dopamine (DA) actions in the spleen. (**A**) DA acts on dopamine receptors (DR) and increases the proliferation of IFNγ-producing splenocytes stimulated with concanavalin A (ConA) or anti-CD3. (**B**) All five DA receptors have been detected in splenic NK cells. D1-like receptors (D1R and D5R), when stimulated by DA, lead to increased cytotoxicity. By contrast, when DA acts on D2-like (D2R, D3R and D4R) receptors, it decreases NK cell-mediated cytotoxicity. Both actions, although opposite of each other, are due to the modulation of the cAMP-PKA-CREB signalling pathway through DA receptors.

The pancreas is an important source of non-neuronal DA and L-DOPA. DA is synthesized in α- and β-cells, which express TH and AADC and regulate glucagon and insulin production in these cells [44]. Rat-derived lines of pancreatic α -cells produce L-DOPA from tyrosine but not DA, while β-cells synthesize DA by the uptake of L-DOPA [45]. DA has an important role in protecting the pancreas and intestinal mucosa from injury, e.g., pancreatitis [46,47,48,49,50,51]. DA-synthesizing enzymes are increased in the pancreas in models of acute experimental pancreatitis [49,50]. DA is an effective treatment for this experimental condition; it reduces the cholecystokinin-induced increase in the expression of inflammatory cytokines (TNFα, IL1β, and IL-6). These effects are D2R-mediated through PP2A-dependent Akt/NF-κB signaling [50]. In other animal models of acute pancreatitis, D2R also attenuates acinar cell necroptosis. In addition, activation of the D2R inhibits oxidative stress-induced macrophage polarization, NF-κB activation, and the NLRP3 inflammasome. Activation of D2R also reduces trypsinogen activation and HSP70 upregulation, thus contributing to its beneficial effects on the disease, suggesting that activation of D2R may be a pharmacological treatment for acute pancreatitis [51]. DA, vesicular transporters, and DA receptors are expressed in the spleen [52]. Indeed, L-DOPA and DA increased the proliferation of splenic lymphocytes that were stimulated with concanavalin A or anti-CD3 but downregulated the number of IFNϒ producing cells [53]. All five DA receptors have been detected in splenic natural killer (NK) cells and modulate their toxicity by regulating the cAMP-PKA-CREB signalling cascade. D1-like receptor activation enhances NK cell cytotoxicity, while activation of D2-like receptors supresses NK cells [54]. In diabetic septic mice, fenoldopam, a D1-like receptor agonist, attenuated hyperglycemia and systemic inflammation through inhibition of p65NF-kB phosphorylation in the spleen [55]. Fenoldopam inhibits TNFα production in splenocytes even at high concentrations of glucose and inhibits p50NF-kB1 phosphorylation and the canonical NF-kB pathway by inhibiting p65RelA, without affecting the non-canonical NF-kB proteins [55] (Figure 2).

#### 1.2.2. Dopamine in the Liver

Hepatocytes are rich in AADC and synthesize DA that may protect the liver from acute injury. In mice exposed to LPS or LPS/d-galactosamine (D-Gal), a model of acute liver injury, treatment with DA or the D2-like receptor agonist rotigotine (D2R > D3R > D4R > D5R > D1R) reduced the number of abnormal histologic lesions, decreased plasma aminotransferases, and increased survival rates [39,56]. DA also suppressed in the liver the production of TNFα induced by LPS/D-Gal and decreased apoptosis in the treated mice [57]; this may also involve the production of reactive oxygen species [58] and α2-adrenergic receptors [59]. A selective D1-like receptor agonist, A68930 (D1R = D5R), suppressed immune cell-mediated hepatitis in mice by inhibition of IL-4 and interferon γ through the PKA pathway [60]. In liver cancer, fisetin, which can act as a D2R agonist, inhibited TGF-β1 secretion and reduced epithelial mesenchymal transition not only by downregulating VEGFR1, p-ERK1/2, p38 and pJNK signaling pathways, but also by inducing apoptosis of liver cancer cells by activating caspase-3, indicating that DA may inhibit the growth of liver cancer [61]. Moreover, DA receptors, specifically D2R, regulate cytochrome P450 (CYP) enzymes in hepatocytes by activation of the pathway insulin/PI3K/AKT [62]. CYP enzymes from the liver are crucial in the metabolism of numerous environmental toxic substances [62]. Thus, DA receptors in hepatocytes may be protective against the toxicity and carcinogenicity of numerous substances (Figure 1).

#### 1.2.3. Dopamine in the Lung

Endogenous DA levels are high in the lungs [63]. The lung expresses AADC and synthesizes DA mainly in alveolar type II epithelial cells and in pulmonary neuroendocrine cells [64]. Pulmonary arteries express both D1R and D2R [65,66,67]; these receptors are also expressed in the carotid bodies of experimental animals [68,69], suggesting that DA has a role in the control of ventilation [70] and that DA in this organ is involved in a number of pulmonary functions, as well as in anti-inflammatory effects (Figure 1). DA may improve respiratory muscle function and has been used for treating conditions such as bronchial asthma and chronic obstructive pulmonary disease to induce bronchodilation because D2R activation decreases pro-inflammatory reflex responses and inhibits neurogenic inflammation [63,71,72]. D1-like receptor stimulation with fenoldopam in macrophages, human monocytes, and alveolar epithelial cells, but not in neutrophils, decreases IL-1β via AMPK activation and reduces in vivo LPS acute lung injury through mechanisms involving inhibition of TLR4 in macrophages and a decrease in paracrine IL-1β-induced adverse signaling in alveolar epithelial cells [73]. However, D1R increases mucus production that can worsen airway obstructive symptoms [74]. However, dopamine has been shown to ameliorate LPS-mediated pulmonary edema and decrease myeloperoxidase activity, an index of neutrophil infiltration that may be related to the D2R [75]. These studies highlight the importance of D1-like and D2-like receptor signaling pathways in reducing acute lung injuries that are related to the inhibition of immune cell activation.

#### 1.2.4. Dopamine in the Cardiovascular System

DA receptors in the heart participate in myocardial hypertrophy and fibrosis. Dopamine receptors are expressed in atrial and ventricular walls, as well as in coronary arteries [76]. In the human heart, D1R, D2R, D4R, and D5R are present in the myocardium and epicardium, but so far there are no reports of D3R expression in this organ [77]. The specific role of each DA receptor in the human heart still needs to be determined. D1R signaling downregulated the NLRP3 inflammasome in cardiomyocytes treated with doxorubicin and reduced cardiac injury and fibrosis in doxorubicin-treated mice by also suppressing the NLRP3 inflammasome in the heart [78]. In cultures of neonatal rat ventricular myocytes, D2-like receptor stimulation with bromocriptine inhibited the angiotensin II-induced hypertrophy of neonatal rat ventricular myocytes [79] and decreased apoptosis in myocytes subjected to ischemia/reperfusion injury [80]. In a rat model of cardiac hypertrophy, bromocriptine decreased the hypertrophy index by acting on the D2R [81]. D2R signaling is involved in the cardioprotective effects of ischemic preconditioning and postconditioning. Ischemia/reperfusion in the heart increased D2R expression; D2R expression was further increased by conditioning and additional treatment with the D2-like receptor agonist bromocriptine, which improved cardiac function, resulting in a more pronounced reduction in apoptosis, cardiomyocyte damage, and myocardial infarct [82,83,84,85]. In healthy C57BL/6J mice, the activation of D3R with pramipexole was capable of reducing cardiac hypertrophy after morphine administration [86]. However, *Drd3*^−/−^ mice had increased interstitial fibrosis and expression of collagen type I which increased with age, indicating that D3R has a role in the development of aging-related remodeling, cardiac fibrosis, and dysfunction in mice [87]. In ex vivo and in vitro experiments, activation of D4R with PD168077 improved cardiac function in the ischemia/reperfusion-injured heart, reduced infarct size, and enhanced the viability of cardiomyocytes impaired by this injury by increasing PI3K and AKT phosphorylation [88].

In endothelial cells, sumanirole, a specific D2R agonist, has antioxidant, anti-inflammatory, and antiapoptotic effects, counteracting those induced by bradykinin, a proinflammatory B2R-activating peptide. The co-stimulation of both B2R and D2R receptors regulated the expression of markers of apoptosis such as Bcl-2, Bcl-xL, and Bax and inhibited the release of IL-6 and endothelin-1 [89]. In human umbilical vein endothelial cells, the D2-like receptor agonist rotigotine (D2R > D3R > D4R > D5R > D1R) inhibited NF-κB activation and reduced its reporter activity, exerting anti-inflammatory actions [90]. DA, receptor not determined, also regulated the interaction of leukocytes and the endothelium, decreasing the transendothelial migration of leukocytes and the expression of ICAM-1 and E-selectin in endothelial cells and attenuating the chemoattractant effect of IL-8 [91,92].

#### 1.2.5. Dopamine in Adipose Tissue

Human adipocytes express four DA receptor subytpes (D1R, D2R, D4R, and D5R), and have been shown to be involved in cytokine/adipokine release, especially during adipogenesis [93,94]. D2R receptors seem to mediate the inhibitory effect of DA on adipocyte prolactin gene expression and release, while D1-like receptors decrease adiponectin, leptin, and IL-6 production and release [93]. However, it also has been reported that D2R activation could upregulate the production of leptin and IL-6 in adipocytes [95]. In that sense, it has been proposed that DA is likely a key signaling molecule connecting adipocytes, immune cells, and sympathetic nerve terminals, playing a role in immune–metabolic diseases such as obesity [94]. The role of DA is not limited to the white adipose tissue. D1-like receptors in brown adipocytes increase oxygen consumption rates and mitochondrial mass through p38 MAPK phosphorylation [96]. Nevertheless, the relevance of the effect of DA and its receptors on brown adipose tissue is still controversial, since the acute peripheral administration of a D1-like receptor agonist SKF38393 in mice only transiently increased brown tissue temperature after injection, while there was no sustained difference in temperature after repeated daily administration [97]. Moreover, these effects could be attributed to indirect consequences of DA receptors activation in the vascular system via peripheral action during administration of DA receptor agonists [98]. Future studies are needed to understand the role of local DA receptors in adipose tissue.

#### 1.2.6. Dopamine in Bone and Connective Tissues

DA reduced the osteolysis induced by titanium particles, as well as the formation of osteoclasts and the expression of genes related to osteoclastogenesis, and alleviates peri-implant osteolysis, a common complication of prostheses implantation. Peri-implant osteolysis is generated by the recruitment of fibroblasts, osteoclasts, osteoblasts, and immune cells that produce significant amounts of cytokines and chemokines, resulting in stimulation of the formation of osteoclasts and bone reabsorption. [99]. The DA effect was reversed by haloperidol, a D2-like receptor antagonist (D4R > D2R = D3R = D1R > D5R), while the D1-like-receptor antagonist SCH23390 had no effect [99]. D2R agonism in mice with collagen-induced arthritis decreases the signs of inflammation and the imbalance of Treg cells. The D2-like receptor agonist quinpirole (D3R = D4R = D2R) decreased the expression of Th17-related cytokines, IL-7 and IL-22, and increased Treg anti-inflammatory cytokines in mice with arthritis induced by collagen [100]. By contrast, mice lacking the D2R presented with more severe limb inflammation, more expression of IL-17 and IL-22, and a downregulated expression of anti-inflammatory cytokines when compared to wild-type mice. However, in mice lacking the D1R, relative to wild-type mice, collagen-induced arthritis did not alter limb inflammation [100].

#### 1.2.7. Dopamine and the Kidney

The kidney produces DA that is not further transformed to norepinephrine. The glomerulus freely filters DA in the plasma, however, the concentration of free DA in the plasma is usually in the low picomolar range [101,102] and cannot contribute significantly to urinary dopamine, the concentrations of which are in the micromolar range [103,104,105,106]. The contribution of renal dopaminergic nerves is less than 30% of the kidney production of DA [103,107,108,109,110,111,112,113]. The main source of renal DA is from the decarboxylation of L-DOPA [114,115,116,117,118,119,120,121]. For the most part, plasma L-DOPA is produced in tissues innervated by the sympathetic nervous system and reflects the turnover of catecholamines [122,123,124,125]. The renal tubules take up L-DOPA from either the circulation or glomerular filtrate and it is converted to DA by AADC [119,120,121]. L-DOPA uptake is mediated by LAT2, an L-type amino acid transporter type 2, and occurs mainly in the proximal tubule; DA production from L-DOPA could not be detected in isolated glomeruli [121]. The renal production of DA relies heavily on how the expression of LAT2 (SLC7A8) is regulated [126,127,128,129].

In the kidney, DA has anti-inflammatory effects. DA decreases the infiltration of monocytes and the expression of IL-6, and improves renal function after transplantation, in brain-dead rats, a condition associated with severe inflammation in end-organ targets [130]. Mice with intrarenal DA deficiency have increased infiltration of inflammatory cells and oxidative stress [131]. Moreover, decreased renal DA production is associated with increased detrimental effects of angiotensin II on renal injury, as well as worsening of diabetic nephropathy [132,133].

The effect of DA on oxidative stress in the kidney has special importance, as the imbalance between the generation of oxidants and antioxidants is a key player in the pathogenesis and progression of hypertension and the development of renal damage [134,135,136,137]. Renal D1R function is impaired by oxidative stress. Inhibition of the redox-sensitive transcription factor nuclear factor E2-related factor 2 in mice resulted in oxidative stress, renal functional impairment, and elevated blood pressure [138,139]. Different NADPH oxidase (NOX) homologs and their respective regulatory subunits are involved in the pathogenesis of essential hypertension and subsequent renal damage [137]. NOX1 overexpressing mice have impaired endothelium-dependent relaxation and reduced NO bioavailability [140], similar to NOX2 overexpression [141]. D2R decreases oxidative stress in part by reducing the renal expression of NOX isoforms and activity of NADPH oxidase by increasing the expression of two antioxidant factors, paraoxonase 2 and sestrin 2 [142,143,144]. D1R and D5R also contribute to the inhibition of renal NADPH oxidase activity and decrease reactive oxygen species production via PKA/PKC cross talk [145,146] and PLD2 [147,148], respectively. The D5R also interacts with peroxredoxin-4 to reduce renal oxidative stress and inflammatory factors in mice [149].

DA and its receptors also interact with components of the renin-angiotensin system (RAS) in the kidney in a reciprocal fashion. Angiotensin II receptor (AT1R) activation can decrease renal DA effects by inhibiting the extraneuronal (e.g., renal tubule cells) uptake of DA [150], while D1R and D3R increase renin secretion by juxtaglomerular cells [151,152]. D1-like and D2-like receptors can cause the downregulation of the AT1R in renal proximal tubule cells [152,153,154,155,156,157]. These interactions in the kidney are particularly important for the regulation of blood pressure, as the impaired interaction of the receptors from both systems may play a role in the pathogenesis of genetic hypertension [154]. Nevertheless, these effects are not restricted to the classical RAS axis, as angiotensin-(1-7) increases intrarenal DA production [158].

The D2R plays an essential role in renal homeostasis. In mice, global D2R deletion increases renal inflammation and blood pressure; apocynin treatment, which decreased oxidative stress, normalized blood pressure but did not affect the expression of inflammatory factors [142]. In uninephrectomized mice, selective renal downregulation of *Drd2*, using siRNA technology for one week, increased the renal expression of proinflammatory factors, injury markers, and blood pressure, while in intact mice the same procedure in only one of the kidneys increased inflammation and markers of renal injury in the treated kidney but did not increase blood pressure [142]. However, long-term (4 weeks) renal-selective silencing of *Drd2* in only one of the mouse kidneys not only increased renal expression of proinflammatory and profibrotic factors but also increased blood pressure and decreased renal function [159]. Renal rescue of *Drd2* function in these mice using retrograde ureteral infusion of adeno-associated virus (AAV9) vectors, carrying *DRD2* cDNA, reduced the expression of pro-inflammatory factors and kidney injury, preserved renal function, and normalized systolic and diastolic blood pressures, thus demonstrating that the primary D2R effect is anti-inflammatory and antifibrotic, and renal damage induced by defective D2R function is the cause and not the result of hypertension [159,160]. The protective effects of the D2R in the kidney were also shown by the renal selective overexpression of the receptor, which mitigated the increase in pro-inflammatory and profibrotic factors, decrease in renal function, and increase in blood pressure in mice subjected to ischemia/reperfusion injury [160].

**Figure 3:**
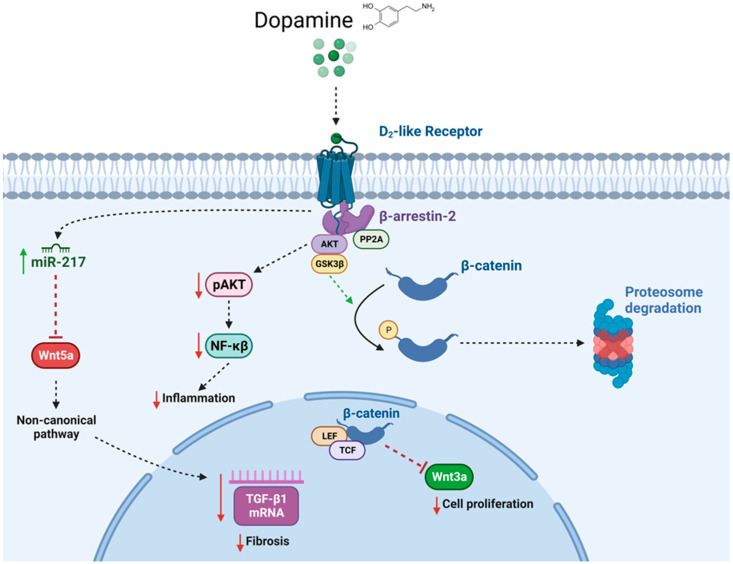
Pathways involved in the protective effect of D2R in the kidney. D2R stimulation, either by G-protein-dependent or G-protein-independent, β-arrestin-2- dependent pathways, increases the expression of miR-217, which downregulates the expression of Wnt5a and by a Wnt non-canonical pathway decreases the transcription of TGF-β1 and results in a decrease in fibrosis. The D2R β-arrestin-2-dependent pathway causes the recruitment of protein phosphatase 2 A (PP2A) and serine/threonine kinase AKT to the β-arrestin-2/D2R complex. PP2A dephosphorylates and inactivates AKT. AKT downregulates the expression of NF-kB and the transcription of a number of inflammatory factors. AKT also regulates through phosphorylation glycogen synthase kinase β (GSK3β), another key signaling kinase. In its non-phosphorylated state, GSK3β is constitutively active, whereas AKT-induced phosphorylation inactivates GSK3β and inhibits β-catenin-driven changes in gene expression, including Wnt3a, resulting in the regulation of cell proliferation.

Specific deletion of the D2R in the renal proximal tubule in male mice increased the expression of pro-inflammatory and profibrotic factors in the kidney, as well as blood pressure [161] (Figure 3). Cells of the renal tubule produce pro-inflammatory and anti-inflammatory cytokines that are secreted across the apical and basolateral membranes of renal tubule cells and contribute to the development and progression of glomerular and tubular injury [162,163,164]. D2Rs are expressed in renal proximal and distal tubule cells. The anti-inflammatory effects of D2R are mediated in part by modulation of the AKT (protein kinase B) pathway [165]. In mouse renal proximal tubule cells, D2R downregulation increases pro-inflammatory factors and NF-kB reporter activity due to the increased phosphorylation and activity of AKT. By contrast, D2R stimulation decreases the expression of inflammatory factors and AKT phosphorylation. Inhibition of protein phosphatase 2A (PP2A) that dephosphorylates AKT reproduces the effects of D2R downregulation, indicating that decreased phosphatase activity is involved in the inhibitory effect of D2R on inflammatory factors [165]. The D2R facilitates AKT inactivation by a G-protein-independent, arrestin-dependent pathway, promoting the formation of a signaling complex with β-arrestin 2, AKT, and PP2A, leading to AKT dephosphorylation and inactivation by PP2A [166]. Glycogen synthase kinase β (GSK3β), a key signaling kinase, is also regulated by AKT through phosphorylation. GSK3β is constitutively active in its non-phosphorylated form, whereas AKT-induced phosphorylation inactivates GSK3β [167]. Active GSK3β phosphorylates β-catenin, a transcriptional co-activator of Wnt, target genes and undergoes proteosomal degradation. However, inactivation of GSK3β activity results in β-catenin translocation to the nucleus and the initiation of the transcription of genes of the Wnt pathway, in particular, Wnt3a [168]. This positive crosstalk between the D2R and Wnt/β-catenin signaling pathways regulates cell proliferation and improves the renal response to injury.

The human *DRD2* gene is highly polymorphic. Several *DRD2* single nucleotide polymorphisms (SNPs), including rs6276 and rs6277, are associated not only with elevated blood pressure and hypertension but also with decreased D2R expression and thus function [169,170,171,172,173]. These polymorphisms are commonly observed with allele frequencies of 0.422 for rs6276 and 0.229 for rs6277 across several populations [174] In renal proximal tubule cells from subjects carrying rs6276 and rs6277 SNPs, the expression of D2R is decreased about 50% when compared with non-carriers [175,176]. In addition, a marked increase in pro-inflammatory and pro-fibrotic factors (inflammatory cytokines and chemokines, macrophage inflammatory protein-1β, NF-kB1A, IL-1F7, IL-10, IL-22, CCl24, and CXCL14) has been associated with the presence of these SNPs [168,175,176]. These cells also present a characteristic phenotype of epithelial-–mesenchymal transition (EMT) with increased TGF-β signaling, a fibrotic pathway, and increased Smad3 and Snail1 transcription factors, resulting in increased collagen I, fibronectin 1, and vimentin synthesis [175]. Transient transfection of a plasmid harboring cDNA of human wild-type *DRD2* negated the pro-inflammatory and pro-fibrotic effects of the SNPs [175,176]. The increase in TGF-β1 is, in part, the result of the positive regulation of the D2R on the expression of miR-217. Cells carrying *DRD2* SNPs have a reduced expression of miR-217, which resulted in increased TGF-β1 by decreasing the miR-217 repressor effect on Wnt5a. Increased expression of Wnt5a and its receptor Ror2 increases the expression of TGF-β1 through the non-canonical Wnt pathway, resulting in EMT [176]. Moreover, reduced D2R expression in human renal proximal tubule cells is associated with increased Wnt3a expression, which then alters Wnt/β-catenin signaling and results in increased cell proliferation [168]. Importantly, SNPs in *DRD2* are associated with chronic kidney disease in Asian Indians with type-2 diabetes [177], which highlights the translational potential of understanding renal D2R signaling and its implications in health and disease.

## 2. Clinical Relevance

### 2.1. Genetics

Essential hypertension is a major consequence of chronic kidney disease (CKD). However, hypertension may cause progressive kidney disease only in genetically susceptible individuals [178,179,180]. Although genetic-linkage analyses and association studies [4,5,181,182] have implicated several loci and candidate genes in the predisposition to CKD, the genes that contribute to genetic susceptibility to this condition are largely unknown.

Kidney disease may result directly from gene mutations that lead to a dysfunctional protein. However, genetic factors may become evident only in the presence of systemic diseases, such as hypertension and diabetes mellitus, and affect the outcome of renal disease. The difference in the susceptibility to disease progression among patients may be explained by polymorphisms in genes that encode proteins, providing renal tissue protection from permanent damage. Identification of novel genetic factors that determine renal disease susceptibility may increase the understanding of the pathogenesis of CKD and the protective effects of endogenous molecules in the kidney may be exploited to counteract the growing incidence of CKD.

*DRD2* SNPs that result in decreased expression are associated with susceptibility to diabetic nephropathy [177]. The analysis of 16 microarray studies deposited in GEO Datasets of CKD patients, controls, and transplanted kidneys with or without injury showed that in 13 out of the 16 studies, *DRD2* expression is lower in patients with CKD or transplanted kidneys with injury and deficient renal function than in patients with no CKD or transplanted kidneys with no evidence of injury (unpublished). Genetic testing can help to identify the individuals at risk and pharmacological treatment tailored to prevent or ameliorate the insult may decrease the prevalence of renal injury and CKD.

More than 90% of the *DRD2* SNPs are located in intronic and regulatory regions and can modify the interaction with miRNA, transcription factors, or ribosomal translation of mRNA [7]. Only a few polymorphisms are located in the coding region and may affect the protein function, structure, or stability; most of these SNPs are predicted to have decreased protein stability and have pathologic effects [183]. Several of the *DRD2* SNPs are associated with a reduction in D2R expression. The frequency of rs1800497, (also known as Taq1 A, allele of the ANKK1 gene, previously reported to be located in the *DRD2*) is about 22% in the Caucasian population. This variation, which results in a 40% reduction in D2R expression in the striatum without affecting receptor affinity [170,171,184,185], is associated with a good weight loss response to naltrexone/bupropion [186]. This *DRD2* variant decreases D2R expression in human renal proximal tubule cells [175] and the Taq1A2A2 genotype is associated with hypertension with decreased iliac and tricep skinfold thickness [169,187,188,189,190] By contrast, the TaqA1 allele frequency is increased in individuals with type 2 diabetes [191].

*DRD2* rs6277 has a frequency of about 50% in the Caucasian population. *DRD2* rs6277 is associated with decreased mRNA stability and translation, reduced DA-induced up-regulation of D2R membrane expression in vitro [172], and lower D2R expression in the cortex and striatum in healthy subjects [192,193,194]. *DRD2* rs6275 and *DRD2* rs6277 are associated with significant increases in blood glucose after controlling for BMI, age, sex, dosage, and type of antipsychotic medication in treated schizophrenics [195]. *DRD2* rs6276 and *DRD2* rs6277, which are also associated with a decrease renal D2R expression and function, may be important in the pathogenesis of inverse salt sensitivity, a state in which blood pressure is increased by a low salt diet. The presence of *DRD2* rs6276/rs6277 decreases the renal proximal tubule plasma membrane expression of D2R and its affinity to a D_2_-like receptor antagonist. The decreased expression of D2R in cells expressing *DRD2* rs6276 can be related to the increased binding of miR-485-5p miRNA to the rs6276 sequence that represses D2R expression [196].

*DRD2* rs6276, rs35608204, and rs1800499 are linked to and/or associated with type 2 diabetes and may be related to a repressed chromatin state in the endocrine pancreas, which is consistent with impaired insulin secretion and glucose intolerance [197]. rs1799732 (also known as −141 C Ins/Del) is located in the promoter region of the *DRD2*, with an allele frequency of about 9% in the Caucasian population [198]. This SNP is associated with decreased mRNA stability and translation, reducing the DA-induced up-regulation of D2R membrane expression in vitro [172]. *DRD2* rs2283265, rs1076560, and 1079727 alter mRNA splicing and transcription process in exon 6, leading to two isoforms of *DRD2*, which are D2 long and D2 short, and is associated with low mRNA expression levels [199,200,201]. More specifically, these SNPs were associated with reduced expression of the receptor in the prefrontal cortex and striatum in healthy subjects and in schizophrenics [202,203,204]. Two *DRD2* SNPs were associated with reductions in *DRD2* activity; rs1801028 is associated with reduced DA affinity [205] and rs1079597 with lower receptor binding [170]. By contrast, rs12364283 is associated with an increase in the D2R protein caused by an increase in mRNA expression levels [204,206]. The roles, if any, of these *DRD2* SNPs in blood pressure regulation and/or renal function are not known.

### 2.2. Effects of Antipsychotics on Systemic Inflammation

There is an increased mortality rate and shortened life expectancy in schizophrenic patients, among other reasons, because of the adverse effects of antipsychotic drugs, most of which are D2R antagonists or partial agonists [207,208]. The risk of acute and chronic kidney disease is increased in schizophrenic patients and also increases with the use of both typical and atypical antipsychotics [209,210]. Moreover, patients treated with several atypical antipsychotics have higher levels of cytokines and chemokines and are at higher risk of acute kidney injury than those treated with typical antipsychotics [211,212,213]. This is not a general finding, as the anti-inflammatory effect of an atypical antipsychotic has also been reported for risperidone with no anti-inflammatory effect of clozapine [214]. However, other studies have shown that clozapine increases the plasma levels of soluble tumor necrosis factors sTNFR1 and TNFR2, while haloperidol has no effects [212]. A population-based study in older adults also showed a greater risk of acute kidney injury in patients prescribed with atypical antipsychotic drugs than those not prescribed with these drugs [215,216]. Furthermore, a meta-analysis of inflammatory biomarkers in healthy volunteers taking both typical and atypical antipsychotics had increased plasma levels of C-reactive protein, IL-6, and TNFα [217].

Schizophrenia, by itself, and treatment with clozapine or olanzapine are associated with weight gain, metabolic risk factors, morbidity, and modifications in cytokines and adipokines that are in part gender dependent [218,219]. Most studies have shown that in these patients, serum adiponectin levels are lower than in controls and are associated with the development of insulin resistance, metabolic syndrome, type 2 diabetes, and risk of cardiovascular events. Other atypical antipsychotics such as risperidone and paliperidone have an intermediate risk, while others such as aripiprazole have less or little effect on body weight. Similarly, typical antipsychotics also carry the potential risk of weight gain [220,221]. One of the potential mechanisms involved in the weight gain induced by antipsychotics is the blockade of D2R and D3R, as antipsychotic drugs that only interact with these receptors can significantly increase weight gain [222,223].

## 3. Conclusions

Dopamine is produced locally in several peripheral organs and different cell types and has autocrine and paracrine effects influencing many organ functions. Its negative effects on oxidative stress and inflammation involve several receptors, and the effects may be tissue- or organ-specific. In most tissues and several immune cells, D2-like receptors have anti-inflammatory and antifibrotic properties, while D1-like receptors anti-inflammatory effects are, for the most part, related to the regulation of oxidative stress and function of infiltrating immune cells. In the kidney, oxidative stress and inflammation are major mediators of the development and progression of disease. Low-grade inflammation is associated with cardiovascular disease. Infiltration of inflammatory cells and increased expression of proinflammatory factors are crucial in the development of renal injury, as well as in the induction and maintenance of hypertension. Human *DRD2* SNPs that decrease expression of the receptor are associated with hypertension and increase the susceptibility to chronic kidney disease. This is related to the loss of the antioxidant and anti-inflammatory effects of renal D2R. The implementation of genetic testing to identify individuals at risk and the tailoring of pharmacological treatment to ameliorate the insult should decrease the consequences of renal injury and, therefore, the prevalence of chronic kidney disease.
